# Feeding behaviour and mortality of *Philaenus spumarius* exposed to insecticides and their impact on *Xylella fastidiosa* transmission

**DOI:** 10.1002/ps.7105

**Published:** 2022-08-17

**Authors:** Clara Lago, Daniele Cornara, Serena Anna Minutillo, Aránzazu Moreno, Alberto Fereres

**Affiliations:** ^1^ Instituto de Ciencias Agrarias (ICA). Consejo Superior de Investigaciones Científicas (CSIC). Calle Serrano 115dpdo Madrid Spain; ^2^ Departamento de Producción Agraria Escuela Técnica Superior de Ingeniería Agronómica, Alimentaria y de Biosistemas (ETSIAAB), Universidad Politécnica de Madrid (UPM) Madrid Spain; ^3^ Department of Soil, Plant and Food Sciences Entomological and Zoological Section, University of Bari Aldo Moro Bari Italy; ^4^ International Centre for Advanced Mediterranean Agronomic Studies ‐ Institute of Bari (CIHEAM‐Bari) Valenzano Italy; ^5^ Associate Unit IVAS (CSIC‐UPM) Control of Insect Vectors of Viruses in Horticultural Sustainable Systems Madrid Spain

**Keywords:** integrated pest management (IPM), electrical penetration graph (EPG), pesticide, transmission, spittlebugs, vector‐borne disease

## Abstract

**BACKGROUND:**

Insecticides are essential, though controversial tools in modern pest management. Insecticides can slow the spread of key vector‐borne plant pathogens, but often lead to inconsistent results given that insecticide use is generally focused on acute toxicity under no‐choice conditions. Here, we analysed the lethal (survival) and sublethal (feeding behaviour) effects of six commercial products (acetamiprid, deltamethrin, spinosad, sulfoxaflor, pyrethrin and kaolin) on *Philaenus spumarius*, vector of the bacterium *Xylella fastidiosa*. Furthermore, we assessed the impact of insecticides displaying different degrees of acute toxicity against spittlebugs (highest to lowest: acetamiprid, pyrethrin and kaolin) on the transmission of *X. fastidiosa* by *P. spumarius* under both free‐choice and no‐choice conditions.

**RESULTS:**

Deltamethrin, acetamiprid and to a limited extent pyrethrin significantly altered the feeding behaviour of *P. spumarius*. Deltamethrin and acetamiprid were highly toxic against *P. spumarius*, but the mortality induced by exposure to pyrethrin was limited overall. By contrast, spinosad, sulfoxaflor and kaolin did not significantly impact *P. spumarius* feeding behaviour or survival. Under no‐choice conditions, both pyrethrin and acetamiprid reduced the *X. fastidiosa* inoculation rate compared with kaolin and the control. On the other hand, pyrethrin reduced transmission, but acetamiprid failed to significantly affect bacterial inoculation under free‐choice conditions.

**CONCLUSION:**

Pyrethrin was the only compound able to reduce *X. fastidiosa* transmission under both free‐choice and no‐choice conditions. *Xylella fastidiosa* management strategy based exclusively on the evaluation of insecticide acute toxicity under no‐choice conditions would most likely fail to prevent, or slow, bacterial spread. © 2022 The Authors. *Pest Management Science* published by John Wiley & Sons Ltd on behalf of Society of Chemical Industry.

## Graphical abstract

Acetamiprid was toxic against spittlebugs, whereas pyrethrin was not. Both compounds altered their feeding and reduced bacterium transmission under no‐choice conditions, but acetamiprid failed to affect transmission under free‐choice conditions.

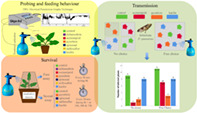

## 1 INTRODUCTION

*Philaenus spumarius* L. (1758) is the only epidemiologically relevant vector of *Xylella fastidiosa* Wells (1987) in Europe.^1^, ^2^ *Xyllela fastidiosa* is the aetiological agent of plant diseases affecting several economically important crops, including Pierce's disease of grapevines, olive quick decline syndrome, almond leaf scorch disease and citrus variegated chlorosis.^3^, ^4^ Since *X. fastidiosa* was detected in Italy in 2013, subsequent mandatory large‐scale surveys throughout Europe have led to its identification in France, Germany, Spain and Portugal.^5^, ^6^ No curative treatment upon infection is available, thus control strategies should prevent bacterium inoculation by the vector, reducing insect abundance and ideally limiting insect access to the host plant.^7^, ^8^, ^9^ However, given that spittlebugs were not considered pests before the detection of *X. fastidiosa* in the Palaearctic realm, limited data are available on control tools effective against these insects, including pesticides.

Modern agriculture relies mainly on pesticide use for crop protection.^10^ However, integrated pest management (IPM) is an alternative to conventional pest control that aims to reduce pest impacts on crops through a combination of ecological and economically sustainable techniques, including the use of pesticides when necessary, to mitigate their non‐target effects.^11^, ^12^ Research on the effects of pesticides on both target and non‐target organisms is pivotal for developing effective IPM strategies integrating pesticides with other control tools.^11^, ^13^ Traditional technical studies are mainly focused on the lethal effects of pesticides while overlooking their impact on insect physiology and behaviour. However, once understood and thoroughly characterized, physiological and behavioural effects might be exploited and integrated into management strategies aimed at interfering with the transmission and spread of vector‐borne plant pathogens.^11^, ^12^, ^14^

To date, the few published studies on insecticide effects on *P. spumarius* have assessed the acute toxicity induced by several molecules under no‐choice conditions. According to Dongiovanni *et al*.,^15^ neonicotinoids and pyrethroids showed the highest efficacy and persistence among the tested compounds against adults of *P. spumarius* and *Neophilaenus campestris* (Fall) (Hemiptera: Aphrophoridae). Moreover, neonicotinoids, specifically imidacloprid, exhibit a strong antifeedant effect against many insect pests.^16^, ^17^, ^18^ However, the recent banning of some neonicotinoids such as imidacloprid for field use in the European Union (EU Commission implementing Regulations 2018/783, 784 and 785) reduces the number of available products for controlling vectors of *X. fastidiosa*. Organophosphate insecticides yielded lower mortality rates or inconsistent results, whereas a good knockdown effect but poor persistence was reported for spinosad by Dáder *et al*.,^19^ who found that pyrethroids were the most effective insecticides against nymphs of *P. spumarius* among several active compounds tested. Nevertheless, given that transmission of *X. fastidiosa* by its vectors may occur within a few minutes upon insects landing on the host plant, bacterial cells could be inoculated into the host plant before even an acutely toxic molecule displays its lethal effect.^20^, ^21^ However, non‐acutely toxic insecticides might impact feeding behavioural activities that lead to pathogen transmission. As an example, kaolin clay interferes with insect host plant recognition, camouflaging the plant with a white coating and affecting the probing and settling behaviour of piercing–sucking insects, such as psyllids, thrips and sharpshooters.^22^, ^23^, ^24^, ^25^ Indeed, kaolin has been demonstrated to reduce the transmission rate of *X. fastidiosa* by *Homadolisca vitripennis* Germar (1821) (Hemiptera: Cicadellinae).^23^, ^26^ Thus, when planning an *X. fastidiosa* control strategy targeting vectors, a thorough evaluation of either lethal or sublethal (physiological and behavioural) effects exerted by a pesticide on the insect vector(s) is of fundamental importance.

Therefore, the objectives of this study were to: (a) evaluate the acute toxicity and effects on feeding behaviour of the meadow spittlebug of different commercially available compounds proposed for vector control in olive; (b) assess the impact of selected compounds with a different degree of acute toxicity on the inoculation of *X. fastidiosa* by *P. spumarius* under no‐choice and free‐choice conditions.

## 2 MATERIALS AND METHODS

### 2.1 Effect of commercial products on the feeding of *Philaenus spumarius*

#### 2.1.1 Insects and plants

Experiments were conducted at the Institute of Agricultural Sciences of the Spanish National Research Council (ICA‐CSIC, Madrid, Spain) from April to June 2020. *Philaenus spumarius* nymphs were collected in a recreational and unmanaged *X. fastidiosa*‐free area in Hoyo de Manzanares, Madrid (Spain) on herbaceous plants: *Sonchus* sp. L., *Eryngium campestre* L., *Carduus tenuiflorus* L. *Taraxacum* sp. (Wigg) and *Borago officinalis* L. Nymphs were reared to adulthood inside BugDorm cages with *Sonchus oleraceus* L. using four‐ to five‐leaf stage plants, and were kept under greenhouse conditions (24:18 ± 2°C day/night temperature, 14:10 h light/dark photoperiod and 60%–70% relative humidity [RH]). Once adults emerged, males and females were kept together inside the cages with no more than 150 insects per cage and were maintained as described for nymphs.

#### 2.1.2 Commercial products

Six compounds authorized in the EU for *X. fastidiosa* vectors with different modes of action were screened (Appendix S1): two insecticides that have shown high acute toxicity against *P. spumarius* and long persistence, acetamiprid and deltamethrin;^15^ one insecticide that induced high mortality but with poor persistence, spinosad;^15^ one product that induced low mortality and poor persistence but being one of the very few organic products recommended for controlling *X. fastidiosa* vectors in the United States, pyrethrin;^15^ one particle film based on clays that affects insect host plant finding ability, kaolin;^25^ and a systemic insecticide reported to be effective against *P. spumarius* nymphs but never tested on adults, sulfoxaflor.^19^ Detailed information for the compounds is shown in Appendix S1. Plants sprayed with tap water were used as a control.

#### 2.1.3 Electrical penetration graph recording

The effect of the six compounds referred to above on the feeding behaviour of *P. spumarius* was monitored using the electrical penetration graph (EPG) technique. All EPG assays were conducted under controlled environmental conditions (23–25°C and 50% ± 10% RH). EPGs were performed on *S. oleraceous* plants, a preferred host plant for *P. spumarius*.^27^ Plants were at the four‐ to five‐leaf stage at the beginning of experiments. The plants were sprayed with the products at the commercial recommended doses 24 h before the onset of the experiments. All compounds were applied using a hand‐sprayer (Matabi Berry® 1.5 L, Goizper) until runoff. Adult *P. spumarius* (1 week to 3 months old) were anaesthetized by applying CO_2_ for 5 s, immobilized with a vacuum device and connected to an EPG device as described previously.^28^ Each individual insect was placed on the adaxial leaf surface, but was free to move to other parts of the leaf. Spittlebug feeding behaviour was monitored continuously for 4 h with three Giga‐4 DC‐EPG and two Giga 8‐DC (EPG Systems) devices placed inside a Faraday cage. EPG data acquisition and analysis were conducted using Stylet+Software for Windows (EPG Systems).

To study *P. spumarius* feeding behaviour, we referred to waveforms described by Cornara *et al*.:^28^ np (non‐probing), C (pathway; stylet tip in plant tissue), R (resting), Xc (xylem contact), Xi (xylem ingestion) and N (interruption within xylem phase). We also took into account the proportion of escaped individuals during the recording time (4 h) (insect no longer on the plant), total probes (stylets inside the plant tissues: C + Xc + Xi + N + R), successful probes (probes where the insect reached the xylem), unsuccessful probes (when the insect was unable to perform any Xi) and sustained probes (probes containing Xi longer than 5 min). We studied the effect of the treatments on non‐sequential EPG variables (number of waveforms events per insect; total waveform duration per insect in minutes; and mean duration of waveforms per insect in minutes), percentage probing time (C + Xc + Xi + N + R) and percentage Xi (time spent in Xi referred to as the total duration of the recording), as well as the sequential variables that are described in Appendix S2. A recording was considered valid and included in the analysis only when the insect performed at least one probe; that is, inserted the stylets into the plant tissues at least once during the recording. Overall, we analysed 18–20 recordings per treatment with a male to female ratio of 1:1. When the number of insects that produced a given waveform (PPW) in a certain treatment was fewer than four, that treatment was not considered in the analysis.

#### 2.1.4 Analysis of EPG data

EPG waveforms were marked manually using ‘Stylet+a’ software (EPG Systems), and behavioural variables were calculated using an Excel macro template designed for xylem feeders. The effect of compounds and sex on the feeding behaviour of *P. spumarius* was analysed using a Kruskal–Wallis test followed by a Steel–Dwass pairwise comparison test. The effect of the compounds and sex on the proportion of escaped individuals was analysed by a chi‐squared test. The analyses were performed for either the total duration of the recording or the hourly trend of EPG variables (1, 2, 3 or 4 h). When studying the effect of the compounds by hours, treatments with fewer than four recordings with alive/non‐escaped insects were removed from the analysis. A Kaplan–Meier survival analysis with log rank Mantel Cox significance test was used to compare the differences in mortality rates between treatments after 4 h of exposure. Statistical analysis was conducted with R software (2020; R Core Team).

### 2.2 Acute toxicity of insecticides on *Philaenus spumarius*

Experiments were conducted during June 2021 in a growth chamber under controlled conditions (23:18 ± 2°C day/night temperature, 16:8 h light/dark photoperiod and 60%–70% RH). The insects and plants used and their maintenance were as described in Section 2.1.1. Plants were treated with the insecticides as explained in Section 2.1.3. All tests were conducted under no‐choice conditions.

#### 2.2.1 Acute toxicity after a short (4 h) exposure period

Spittlebugs were caged in a plastic mesh cylinder for 4 h on *S. oleraceous* plants previously treated with the chemical compounds. Plants were also treated as explained above with the six compounds (deltamethrin, acetamiprid, pyrethrin, spinosad, sulfoxaflor, kaolin) and with tap water (untreated control plants). Each experimental unit consisted of three individual insects of the same sex transferred to one treated plant (3 insects per plant and 14 plants per treatment). The number of dead/alive insects was assessed every 30 min up to 4 h.

#### 2.2.2 Acute toxicity after a long (72 h) exposure period

In accordance with the results obtained on the survivorship of spittlebugs after 4 h of exposure to the six compounds, an additional assay was conducted to assess the effect of selected compounds on the survival of *P. spumarius* after longer exposure periods: 24, 48 and 72 h. The compounds selected for this assay were spinosad, sulfoxaflor, kaolin, pyrethrin and a tap water control. Acetamiprid and deltamethrin caused 100% mortality within 2 h upon insect caging on treated plants (see Results section); thus, both pesticides were discarded for this long‐exposure assay. The number of dead/alive individuals was checked every 30 min for 4 h and after 24, 48 and 72 h. Each experimental unit consisted of ten adults caged (1:1 sex ratio) on one *S. oleraceus* plant treated and maintained as explained in Section 2.2.1 (ten insects per plant, six plants per treatment).

#### 2.2.3 Data analysis of mortality

A Kaplan–Meier survival analysis with log rank Mantel Cox significance test was used to compare the differences in mortality rates among groups. Statistical analysis was conducted with R software (2020; R Core Team).

### 2.3 Effect of insecticides on inoculation of *Xylella fastidiosa* by *Philaenus spumarius*

Given our EPG results, only three compounds were selected for the *X. fastidiosa* transmission assays: (a) acetamiprid, a systemic insecticide that shows a high mortality rate, significant disruption of probing and feeding behaviour, and a strong repellent effect; (b) pyrethrin, which induces low acute toxicity and has a low impact on feeding compared with acetamiprid, but a moderate repellence; (c) kaolin, which exhibits neither acute toxicity nor repellent effects and does not affect the spittlebug feeding behaviour activity but interferes with the host finding ability of sharpshooters in grapevines, thus reducing the spread of Pierce disease;^26^ and (d) water, which was used as a control. Recipient plants were 3‐month‐old periwinkle (*Catharanthus roseus* (L.) G. Don) seedlings grown in a greenhouse under controlled conditions (25 ± 1°C, 60% RH) inside 1‐L pots filled with a basal layer of expanded clay and a mix of soil, peat and pumice (2:3:1), and watered three times per week. *Philaenus spumarius* individuals were collected from olive orchards infected with *X. fastidiosa* in Gallipoli (Southern Italy) during summer 2020 and reared on sunflower (*Helianthus annus*) plants inside BugDorms in an insect‐proof chamber under controlled conditions (26 ± 3°C, 40% RH). Ten days before each test, spittlebugs were transferred to a *X. fastidiosa*‐infected olive plant to maximize the infectivity. We conducted two types of inoculation tests: (a) a no‐choice assay in which infective *P. spumarius* were caged for an inoculation access period (IAP) of 72 h on periwinkle plants all sprayed with one of the three products or a water control; and (b) a free‐choice assay in which infective *P. spumarius* were released in a cage containing plants treated with the different compounds arranged together for an IAP of 72 h. Twenty‐four hours before releasing the spittlebugs into the cages, plants were treated with the compounds under screening (and water for control) using a hand‐sprayer (Matabi IK, 1.5 L) until runoff. We used an aphid‐proof net and plastic cylindrical cages (80 L). Each cage housed eight plants and three spittlebugs were released per plant, giving 24 insects per cage. For the no‐choice test, we used three cages per treatment (24 plants and 72 spittlebugs per treatment). For the free‐choice experiment, we performed 11 replicates (a total of 11 cages, 8 plants per cage, 2 plants per treatment in the same cage, giving 22 plants per treatment). All replicates were performed in parallel on the same date. In this second trial with mixed treatments in the same cage, the position of plants treated with the same compound was switched in each cage to avoid positional effects. At the end of the IAP, spittlebugs were collected. The number of alive, dead and missing individuals was recorded. Spittlebugs were stored in 75% ethanol at −20°C and subject to testing for *X. fastidiosa* using real‐time quantitative PCR (rt‐qPCR).^29^ Upon insect collection, periwinkle recipient plants were sprayed with fungicides and insecticides and stored in an insect‐proof chamber under controlled conditions (25 ± 2°C, 55% RH). Plants were tested for *X. fastidiosa* 3 months after the IAP by rt–qPCR.^30^ Periwinkle plants found wilted at the end of the 3‐month incubation period were discarded and not analysed for *X. fastidiosa*. Nine healthy periwinkle plants were used as a negative control. The plants were positioned out of the cages during the IAP, stored together with the recipient plants during the incubation period, and finally tested for *X. fastidiosa* by rt–qPCR.^30^

#### 2.3.1 Data analysis of inoculation tests

The effect of the insecticides and of *P. spumarius* infectivity (percentage of insects testing positive for *X. fastidiosa* by rt‐qPCR) on the fastidious bacterium inoculation rate (plant infectious status) in the no‐choice and free‐choice assays was analysed with a linear model. Data were transformed, when necessary, with ln(*x* + 1) or √(*x*) to reduce heteroscedasticity and improve normal distribution. A Tukey test was performed for pairwise comparisons. Treatment‐related differences in spittlebugs survival was assessed by Kruskal–Wallis rank sum test. All analyses were run in R (2020; R Core Team).

## 3 RESULTS

### 3.1 Effects of insecticides on feeding behaviour of *Philaenus spumarius*

**FIGURE 1:**
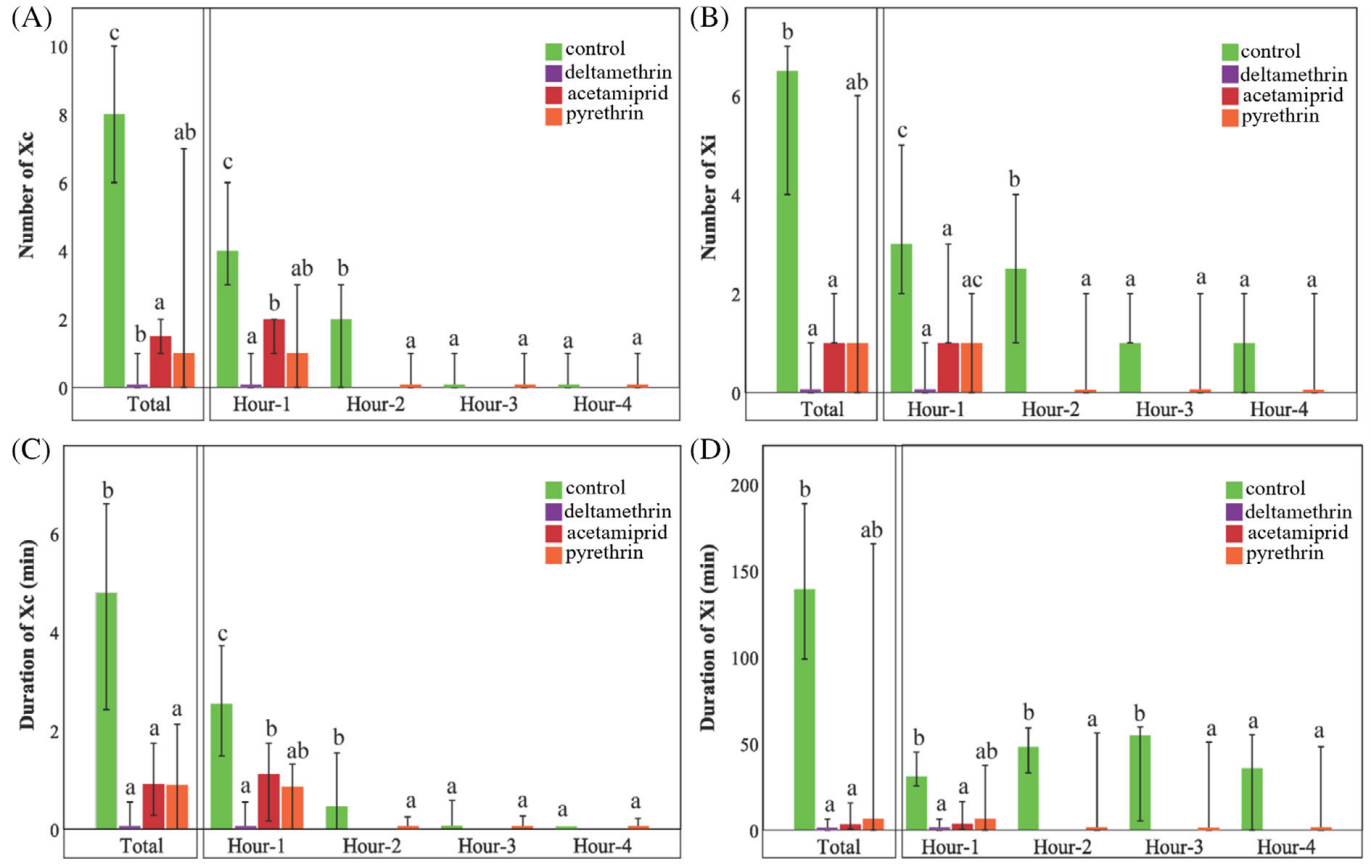
Feeding behaviour of *Philaenus spumarius* after 1, 2, 3 and 4 h of exposure to insecticide treatments and water control. Sulfoxaflor, spinosad and kaolin did not impact any of the variables shown compared with the control; thus, these treatments are not shown. Deltamethrin and acetamiprid were removed from the analysis in hours 2, 3 and 4. The waveform on the *y*‐axis is represented by the median. Error bars are also shown. Different letters indicate significant differences in the values of specific parameters among spittlebugs on treated plants. (A–D) Impact of the treatments on: (A) number of xylem contact (Xc) events per insect; (B) number of xylem ingestion (Xi) events per insect; (C) total duration of Xc per insect; and (D) total duration of Xi per insect.

**FIGURE 2:**
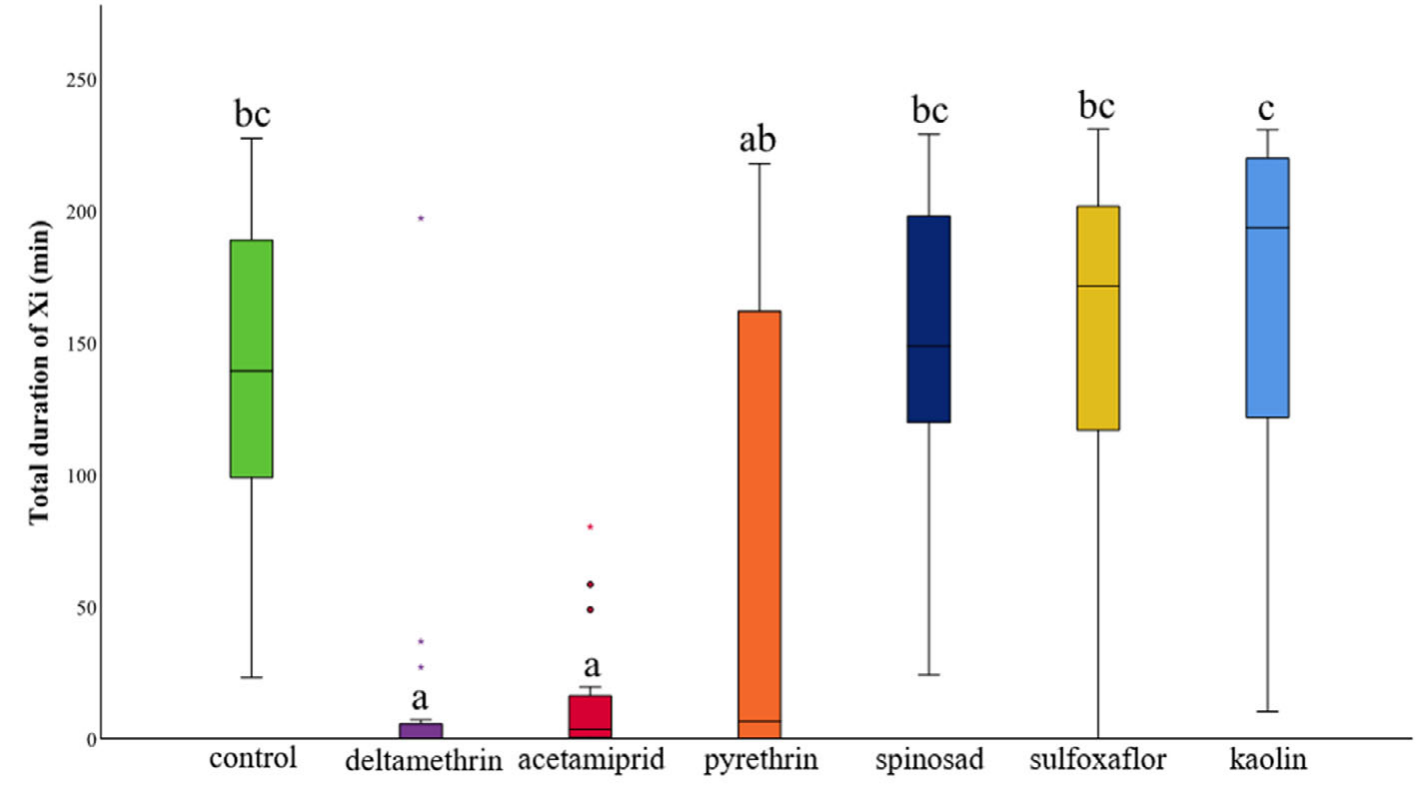
Impact of the insecticide treatments on the total duration of xylem ingestion (Xi). Letters indicate significant differences in the values of specific parameters among the treatments. The horizontal black lines represent median values. Boxes extend from the 25th to the 75th percentile of each group's distribution of values, and vertical extending lines indicate the range of values.

The proportion of escaped insects was significantly increased in plants treated with deltamethrin and acetamiprid compared with the control and most of the treatments (Appendix S3). The number of escaped insects observed on pyrethrin‐treated plants was not significantly different from either control or acetamiprid (Appendix S3). A high impact on several of the EPG variables considered was noted for spittlebugs that fed on plants treated with either deltamethrin or acetamiprid, whereas a moderate impact was observed when the individuals were given access to plants treated with pyrethrin. Indeed, although acetamiprid, deltamethrin and pyrethrin had significant effects on some of the EPG variables, it is worth noting that the differences relative to the control were much larger for plants treated with acetamiprid and deltamethrin than for pyrethrin. By contrast, sulfoxaflor, spinosad and kaolin had no significant impact on *P. spumarius* feeding behaviour. The percentage of probing time and Xi (referring to the total recording time) was significantly reduced on plants treated with deltamethrin and acetamiprid compared with the control (Appendix S3). Insects displayed a significant reduction in the number of successful probes, sustained probes (probes with an Xi longer than 5 min) and Xc (Figure 1A) when fed plants treated with deltamethrin, acetamiprid or pyrethrin compared with the control (Appendix S3). In addition, the PPW (proportion of insects that produced a given waveform) for Xi was 7/19 for insects fed on deltamethrin, 12/19 for pyrethrin and 16/20 for acetamiprid (Appendix S3). By contrast, the PPW for Xi was 18/18 for insects fed on control plants, 19/19 for kaolin, 18/18 for spinosad and 17/18 for sulfoxaflor (Appendix S3). In addition, the number of Xi events was reduced when insects fed on plants treated with deltamethrin and acetamiprid compared with the control, but no significant differences were noted between pyrethrin and the control (Figure 1B; Appendix S3). Spittlebugs offered plants treated with deltamethrin, acetamiprid and pyrethrin also displayed a significantly reduced total duration of the probes (Appendix S3), pathway C (Appendix S3) and Xc (Figure 1C; Appendix S3) compared with the control. In addition, deltamethrin and acetamiprid affected the cumulative duration of the xylem phase (Xc + Xi) (Appendix S3) and the total duration Xi (Figures 1D and 2; Appendix S3). Finally, acetamiprid reduced the mean duration of Xi per insect (Appendix S3).

Regarding the sequential variables, acetamiprid and deltamethrin significantly increased the time from the first np to the first sustained Xi, the time from the first np to the first probe with sustained Xi and the time from the first C to the first sustained Xi compared with the control (Appendix S3). Sex had a significant effect with regard to the time from the first np to the first probe with sustained Xi (*F* = 6.49, df = 1, *p* = 0.012) and the time from the first np to the first probe with Xi (*F* = 3.97, df = 1, *p* = 0.049). However, the interaction between treatment and sex was not significant; thus, the data from both sexes were pooled for analysis (Appendix S3). The remainder of the reported effects of the compounds on meadow spittlebug trophic activity were not influenced by sex (Appendix S3). Of the total 131 recordings, the resting (R) waveform was found in only nine recordings; thus, this variable was not considered in the statistical analysis. The differences observed between treatments related to np and interruption within xylem phase (N); the remainder of the sequential variables are shown in Appendix S3.

We also analysed the EPG recordings based on time (h) to characterize the effect of the spittlebug feeding behaviour trends. Overall, when insects were offered plants treated with pyrethrin, a modification in their feeding behaviour was noted at the beginning of the recording, but this tended to disappear over time. The proportion of escaped insects during the first hour was significantly greater in plants treated with pyrethrin than in the control. This escape effect was no longer observed during the second, third and fourth hours (Appendix S4). The proportion of escaped insects was significantly higher in plants treated with acetamiprid and deltamethrin compared with the remainder of the treatments during all 4 h of the recording (Appendix S4). Because of the high number of escapes from plants treated with deltamethrin (18/19) or acetamiprid (15/20) in the first hour, these two treatments were not considered for further analysis of the EPG variables in the subsequent hours. The effect of the treatments on the number of Xc and Xi and the total duration of Xc and Xi by hours is shown in Figure 1. No significant differences were noted in any of these variables for insects exposed to sulfoxaflor, spinosad or kaolin compared with the control; thus, these treatments are not shown in Figure 1.

In the first hour of recording, the number and duration of Xc were significantly lower on plants treated with deltamethrin, acetamiprid and pyrethrin compared with the control (Figure 1A,C; Appendix S5). Furthermore, during the first hour, the number and duration of Xi were significantly lower on plants treated with deltamethrin and acetamiprid compared with the control, but did not differ significantly from those obtained with pyrethrin (Figure 1B,D; Appendix S5). During the second hour, the number and duration of Xc and Xi were significantly lower when insects fed on plants treated with pyrethrin compared with the control (Figure 1A–D; Appendix S5). In the third hour, no significant differences were observed in the number of Xc and Xi or the duration of Xc between pyrethrin and the control (Figure 1A–C; Appendix S5). By contrast, in the third hour, the duration of Xi was reduced significantly for insects that fed on plants treated with pyrethrin compared with the control (Figure 1D; Appendix S5). Finally, in the fourth hour, no significant differences were observed in the number or the duration of Xc or Xi between pyrethrin and the control (Figure 1A–D; Appendix S5). The differences observed between all the treatments related to the remainder of the EPG variables by hours are shown in Appendix S5.

### 3.2 Acute toxicity of insecticides on *Philaenus spumarius* after a short (4 h) and a long (72 h) exposure period

#### 3.2.1 Acute toxicity after a short (4 h) exposure period

**FIGURE 3:**
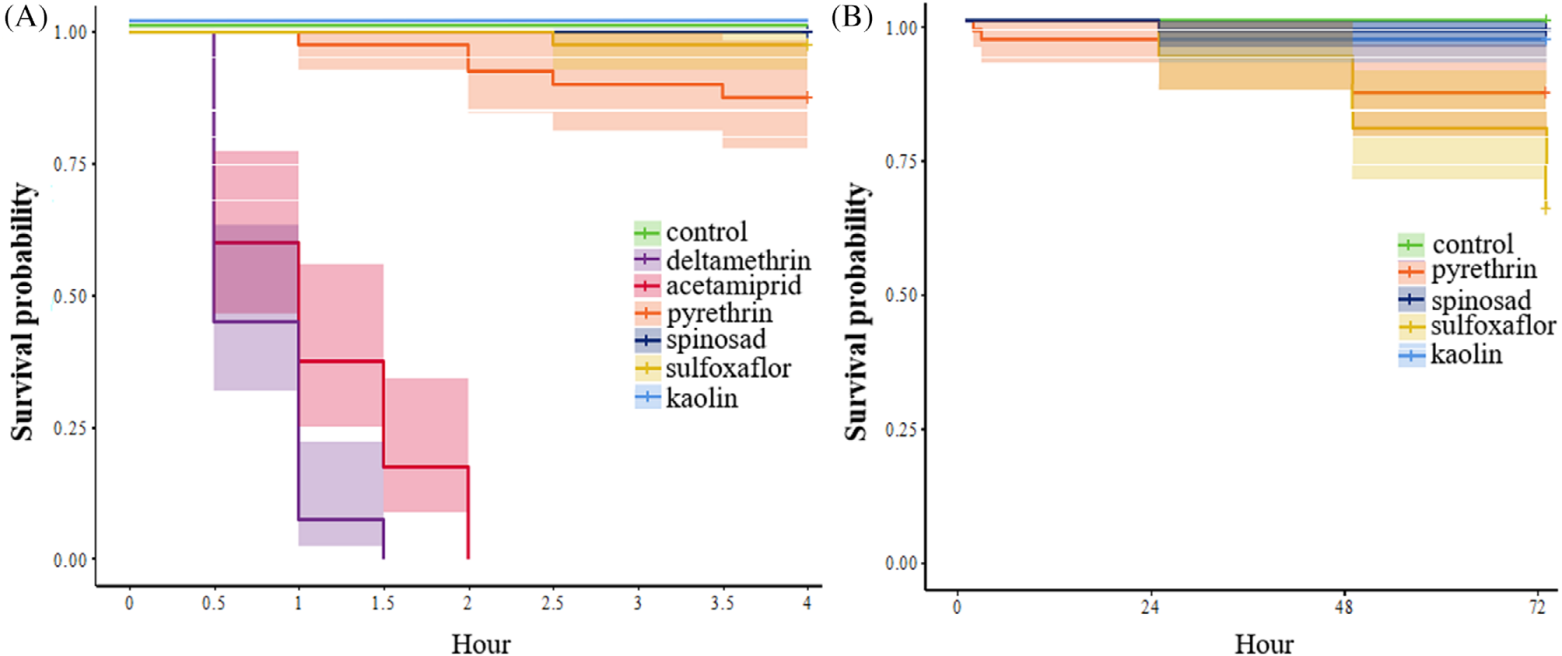
Kaplan–Meier survival curves of *Philaenus spumarius* exposed to different insecticide treatments and water control. The shadowed band surrounding a line of the same colour is the confidence interval. (A) Survival of *Philaenus spumarius* after 4 h of exposure to plants treated with all the treatments (control, acetamiprid, deltamethrin, pyrethrin sulfoxaflor, spinosad, kaolin). Evaluations were performed every 30 min. (B) Survival of *Philaenus spumarius* after a long period of exposure (up to 72 h) to the control, pyrethrin sulfoxaflor, spinosad and kaolin. Evaluations were performed every 30 min for 4 h and 24, 48 and 72 h thereafter.

High acute toxicity against the meadow spittlebug was observed for deltamethrin and acetamiprid with 100% mortality after 1.5 and 2 h of exposure, respectively (Figure 3A). Low mortality was observed among insects exposed to pyrethrin with 12.5% mortality after 4 h (Figure 3A). No insects died after 4 h of exposure to plants treated with kaolin, sulfoxaflor, spinosad or water (control) (Figure 3A).

#### 3.2.2 Acute toxicity after a long (72 h) exposure period

Deltamethrin and acetamiprid treatments were excluded from this assay because 100% of insects died within 2 h of exposure. The mortality at the end of the assay was greater for insects exposed to plants treated with sulfoxaflor and pyrethrin compared with the control, kaolin‐ and spinosad‐treated plants (Figure 3B). The highest mortality over 72 h (approximately 35%) was observed for individuals exposed to sulfoxaflor‐treated plants, followed by those exposed to pyrethrin‐treated plants (approximately 13.3%), which is noticeably much lower than the 100% mortality observed on insects exposed to deltamethrin‐ and acetamiprid‐treated plants for 2 h in the previous assay (Figure 3B).

### 3.3 Effect of insecticides on the inoculation rate of *Xylella fastidiosa* by *Philaenus spumarius*

#### 3.3.1 No‐choice assay

**FIGURE 4:**
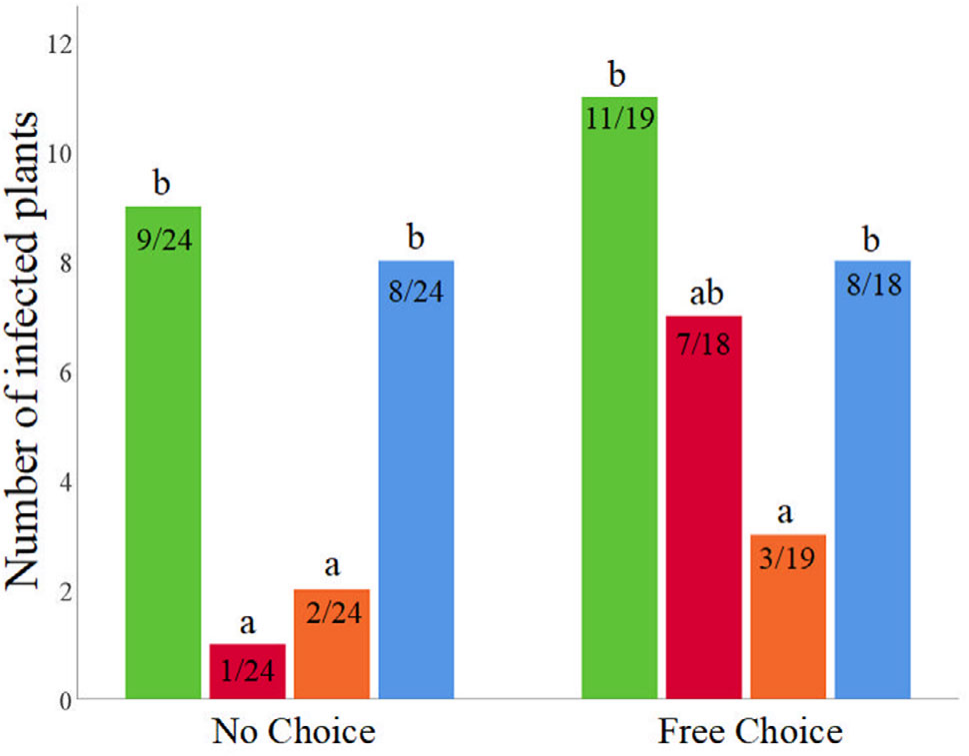
Number of periwinkle plants tested positive for *Xylella fastidiosa* obtained from the transmission assays with infective *Philaenus spumarius*. Plants were previously treated with acetamiprid, pyrethrin, kaolin and water control. Plants were exposed to both no‐choice and free‐choice conditions. Groups accompanied by different letters indicate significant differences.

The nine periwinkle plants used as controls tested negative for *X. fastidiosa* by rt‐qPCR. As shown in Figure 4, in the no‐choice assay, the inoculation rate was reduced significantly on plants treated with acetamiprid (only 1 of 24 plants became infected with *X. fastidiosa*) and pyrethrin (2 of 24) compared with kaolin (8 of 24) and the control (9 of 24) (*F* = 4.59, df = 3, *p* = 0.004). The survival rate was significantly different among the treatments (Kruskal–Wallis *X* ^*2*^ = 64.82, *p* < 0.001). No alive insects were retrieved from plants sprayed with acetamiprid after 72 h of IAP, and approximately 60% mortality was noted for spittlebugs on plants treated with pyrethrin. The highest survival of *P. spumarius* was observed on plants treated with kaolin (66.66%) and on the control (68.05%). There was no effect of spittlebugs infectivity (the percentage of *P. spumarius* testing positive for *X. fastidiosa* by rt‐qPCR) on the inoculation rate (*F* = 0.0001, df = 1, *p* = 0.99).

#### 3.3.2 Free‐choice assay

A different trend was observed for the free‐choice assay (Figure 4). The effect of treatment on the inoculation rate was marginally non‐significant (*F* = 2.552, df = 3, *p* = 0.062). However, considering the pairwise comparison results, plants treated with pyrethrin displayed an inoculation rate significantly lower than control (3 of 19 in pyrethrin, and 11 of 19 in control tested positive for *X. fastidiosa* [*t* = 2.723, df = 69, *p* = 0.03]). By contrast, inoculation rate did not differ significantly among the other treatments (acetamiprid, 7 of 18; kaolin, 8 of 18; control, 11 of 19). Similar to the no‐choice assays, inoculation rate was not affected by spittlebugs infectivity (*F* = 0.267, df = 3, *p* = 0.606).

## 4 DISCUSSION

Pesticides are a key component of pest management strategies used worldwide; however, their application that aims at reducing the spread of vector‐borne plant pathogens often leads to inconsistent results.^31^, ^32^, ^33^ In such a context, an in‐depth characterization of the insecticides’ impact on the vector of a plant pathogen beyond mere observation of their acute toxicity under no‐choice conditions is pivotal to understand their possible impact on disease spread. Specifically, the effect of the pesticide on aspects of paramount importance in the transmission of a plant pathogen, such as host search, settling and feeding behaviours by the insect vector, should be carefully evaluated. In addition, transmission trials on treated plants are crucial given that a strong acute toxicity displayed by a product against the vector is not directly translatable into a reduction of acquisition/inoculation rates.

Therefore, in this work, we evaluated the effect of six insecticides on *P. spumarius* survival and feeding behaviour, and of three of the products on the inoculation rate of *X. fastidiosa*. Acetamiprid and deltamethrin were fast‐acting compounds, inducing 100% mortality within 2 h after exposure of spittlebugs to treated plants, a value significantly greater than that noted for the remainder of the compounds used in our screening, including pyrethrin (Figure 3A). Regarding sulfoxaflor, Dáder *et al*.^19^ reported that this compound was highly effective against meadow spittlebug juveniles. The relative inefficacy observed in our study against adults (65% of insects alive after 72 h) suggests that this pesticide should be used only in the early stages of *P. spumarius* development. Regarding spinosad, our results are not consistent with the efficacy (at high application volumes) of this pesticide against the spittlebug reported by Dongiovanni *et al*.^15^ The differences observed between the two studies could be related to differences in the experimental design between Dongiovanni *et al*.^15^ (spittlebugs exposure to treated olives under field conditions) and our tests (exposure to treated herbaceous plants under lab conditions) or to the building up of resistance by *P. spumarius* populations to spinosad. Banazeer *et al*.^34^ observed a high level of resistance development to spinosad in *Phenacoccus solenopsis* (Hemiptera: Pseudococcidae), whereas other authors have described a resistance to spinosad in insect species of different orders.^35^, ^36^, ^37^, ^38^ The meadow spittlebug has only one generation per year and was not considered a pest in Europe until the outbreak of *X. fastidiosa* in 2013. Moreover, insects in this study were collected from unmanaged habitats; thus, resistance is unlikely, although such a hypothesis should not be discarded.

Given that spittlebugs are strong jumpers^39^ and manipulation during EPGs may be a stress factor, several spittlebugs jumped off the leaves during the EPG recording in all treatments. Nevertheless, the proportion of escaped/no longer on the plant insects during the 4‐h EPG recording period was greater on plants treated with deltamethrin and acetamiprid compared with other treatments. These results are consistent with those observed in our survival trials and seem to be related to the high toxicity of neonicotinoids and pyrethroids observed previously.^15^, ^19^, ^40^ Although pyrethrin seems to be mildly acutely toxic against *P. spumarius* adults, it exhibits a repellent effect in the first hour of exposure with a higher proportion of escaped insects compared with the control (Appendix S4).

In addition to the repellent effect of the insecticides, when analysing the complete EPG recordings (4 h), we observed that acetamiprid and deltamethrin had strong deterrent effects on the feeding behaviour of spittlebugs (for example, reduction of number of Xc and Xi events and their duration), whereas pyrethrin's impact was reduced compared to the former (for example, reduction in Xc and probing time). Similar results were found by Waqas *et al*.^41^ on *Phenacoccus solenopsis* (Hemiptera: Pseudococcidae), which exhibited shorter xylem and phloem ingestions on tomato plants treated with acetamiprid. In addition, *Cacopsylla pyricola* Foerster (Hemiptera: Psyllidae) showed shorter phloem ingestions on plants treated with deltamethrin.^42^ Regarding pyrethrin, it was also observed that the effect of this pesticide was more evident during the first hours of the recordings (fast repellent effect); that is, insects exposed to pyrethrin exhibited a reduction in the number of Xc and Xi events and their duration during the first 2 h. A reduction in the probing frequency on plants treated with pyrethrin compared with the control was previously observed on *Aleurocanthus spiniferus* whiteflies.^43^ Moreover, the antifeedant activity of pyrethrins was previously reported on other hemipterans, and this effect is known to decrease over time.^44^ Nevertheless, the impact of pyrethrin on *P. spumarius* feeding behaviour and its repellent effect tend to disappear in a few hours, possibly as a result of pyrethrin photolability.^45^ The insecticide used in our trials was likely more effective and persistent than that noted under field conditions given that our plants were not directly exposed to the sun but to artificial light. In addition, the activity of detoxifying cytochrome P450, which helps in the metabolism of pyrethrins, could create insect tolerance to this pesticide during ingestion.^46^, ^47^ Indeed, despite antifeedant effects have been well documented for sublethal doses of multiple insecticides,^16^, ^17^ the epidemiological relevance of this effect may be limited, considering the large differences in acute mortality observed for acetamiprid/deltamethrin compared with pyrethrin and the shorter residual efficacy of pyrethrin.

We also explored the direct implications of compound toxicity and impact on spittlebug feeding behaviour: acetamiprid (highly toxic and a feeding deterrent), pyrethrin (moderately toxic and a feeding deterrent) and kaolin (neither toxic nor a feeding deterrent). *Xylella fastidiosa* transmission rate was significantly reduced on pyrethrin‐treated plants under both free‐choice and no‐choice conditions (Figure 3). On the other hand, acetamiprid only succeeded in affecting transmission in the no‐choice tests, while not diverging from the control when spittlebugs were offered a choice among the different treatments. The increase in the inoculation rate observed on acetamiprid‐treated plants under free‐choice conditions may be due to the close presence of plants treated with other compounds. Such an experimental setup may allow *P. spumarius* to visit plants treated with other compounds and avoid the lethal effects of acetamiprid while engaging in more Xc during the duration of the test. Indeed, although not as substantial, inoculation rates were at least modestly higher for all four treatments in the free‐choice trials (Figure 3), which is consistent with this hypothesis.

Moreover, according to the EPG results, it was observed that the proportion of insects that performed Xc and Xi events was greater for insects exposed to acetamiprid, 17/20 and 16/20 respectively, compared with insects exposed to pyrethrin (12/19) for both Xc and Xi. These results suggest that it is possible that during labial contact, the insect could detect the pyrethrin with the sensilla located in the distal part of the labium and be less prone to probe plants treated with pyrethrum when not forced to feed on it.^48^ Thus, once in a cage with different treatments, *P. spumarius* will initially test all the plants, including those treated with acetamiprid, and then likely move to the control and kaolin‐treated plants. We did not investigate the effects of different insecticides on *X. fastidiosa*. However, given that inoculation of the bacterium occurs during the first minutes of a probe,^20^, ^21^ this initial tasting to evaluate host suitability is sufficient for bacterial inoculation to occur on acetamiprid. In addition, it is also possible that a systemic toxicant, such as acetamiprid, would cause problems in phagostimulation, thus more frequently triggering inoculation behaviour, as previously hypothesized by Cornara *et al*.^20^ It is worth noting that in a field setting it would be unlikely that insects freely move back and forth between olive or other susceptible plants treated with different chemical compounds and avoid the lethal effects of acetamiprid. Thus, we believe that the results we obtained under the no‐choice conditions are as equally valid as those found under free‐choice conditions.

In our study, the effect of the different compounds on *X. fastidiosa* acquisition was not evaluated. However, the reduced probing frequency and number and duration of Xc and Xi events observed on plants treated with deltamethrin, acetamiprid and pyrethrin suggest that these insecticides might reduce the opportunity for acquisition to occur, with the latter being a matter of the probability of the spittlebug probing a vessel colonized by the bacterium.^20^, ^49^ However, this hypothesis needs to be tested with specific assays conducted on different host plant/vector combinations. Finally, it should be mentioned that the assays were performed with herbaceous plants rather than economically importance crops (for example, grapevines or olive trees). Concerning feeding behaviour assays, our approach was to use the most preferred plant species to detect any disruption in the feeding behaviour when exposed to plants treated with insecticides, thus *S. oleraceus* were selected.^27^ Similarly, in the transmission assay we used periwinkle, because it has been used successfully as an indicator plant to analyse *X. fastidiosa* transmission in previous studies.^9^, ^50^ Nevertheless, further studies with economically important crops should be also performed.

## 5 CONCLUSION

The *X. fastidiosa* containment strategy should not be based on the exclusive evaluation of the acute toxicity of insecticides under no‐choice conditions, but transmission tests, such as those described above, are essential to validate insecticide effectiveness for reducing disease spread. IPM strategies against *P. spumarius* informed by data gathered through holistic investigations of the insecticide–insect–plant–bacterium interaction should be implemented to control the spread of *X. fastidiosa* diseases in areas where the bacterium is present.

## CONFLICT OF INTEREST STATEMENT

The authors do not have any conflict of interest to declare.

## Supporting information


**Appendix S1.** Supporting information

**Appendix S2.** Supporting information

**Appendix S3.** Supporting information

## Data Availability

The data that support the findings of this study are available in the supplementary material.
