# Supplementary material for: Feeding behaviour and mortality of Philaenus spumarius exposed to insecticides and their impact on Xylella fastidiosa transmission
Source: Pest Manag Sci. 2022 Aug 17;78(11):4841–9. doi: 10.1002/ps.7105 (PMC9804339; doi:10.1002/ps.7105)
Supplement: Supplementary file 2 — Appendix S2. Supporting information [file PS-78-4841-s002.docx]

**SUPPORTING INFORMATION LEYENDS**

**S1.** Description of the commercial products tested. Active ingredient, family, mode of action, commercial product, applied dose (MLD=maximum label dose a) and toxic effect.

**S2.** Description of the sequential variables analysed in the EPG assays.

**S3.** *Philaenus spumarius* feeding behaviour on treated *Sonchus oleraceus* plants. EPG variables presented by mean ± SE based on treatment and PPWs (proportion of individuals producing the waveform type) are also shown. Letters in rows indicate significant differences in the values of specific parameters among the treatments (Kruskal–Wallis analysis and Steel-Dwass post hoc test).

**S4.** The proportion escaped spittlebugs after 1, 2, 3 and 4 h of exposure to plants treated with different compounds during the EPG assays. Comparisons between treatments at different hours were performed by Chi-squared tests. Different small letters indicate significant differences in the values of specific parameters among spittlebugs on treated plants.

**S5.** *Philaenus spumarius* feeding behaviour on treated *Sonchus oleraceus* plants based on hours. EPG variables presented by mean ± SE based on treatment and PPWs (proportion of individuals producing the waveform type) are also shown. Letters in rows indicate significant differences in the values of specific parameters among the treatments (Kruskal–Wallis analysis and Steel-Dwass post hoc test).
